# Modulation of Cell Death Pathways by Hepatitis C Virus Proteins in Huh7.5 Hepatoma Cells

**DOI:** 10.3390/ijms18112346

**Published:** 2017-11-06

**Authors:** Olga V. Masalova, Ekaterina I. Lesnova, Pavel N. Solyev, Natalia F. Zakirova, Vladimir S. Prassolov, Sergey N. Kochetkov, Alexander V. Ivanov, Alla A. Kushch

**Affiliations:** 1 Ivanovsky Institute of Virology, Gamaleya National Research Center of Epidemiology and Microbiology, Ministry of Health of the Russian Federation, Moscow 123098, Russia; wolf252006@yandex.ru (E.I.L.); vitallku@mail.ru (A.A.K.); 2 Engelhardt Institute of Molecular Biology, Russian Academy of Sciences, Moscow 119991, Russia; solyev@gmail.com (P.N.S.); nat_zakirova@mail.ru (N.F.Z.); prassolov45@mail.ru (V.S.P.); kochet@eimb.ru (S.N.K.)

**Keywords:** hepatitis C virus, hepatoma Huh7.5 cells, apoptosis, autophagy, necrosis, caspase, replicon

## Abstract

The hepatitis C virus (HCV) causes chronic liver disease leading to fibrosis, cirrhosis, and hepatocellular carcinoma. HCV infection triggers various types of cell death which contribute to hepatitis C pathogenesis. However, much is still unknown about the impact of viral proteins on them. Here we present the results of simultaneous immunocytochemical analysis of markers of apoptosis, autophagy, and necrosis in Huh7.5 cells expressing individual HCV proteins or their combinations, or harboring the virus replicon. Stable replication of the full-length HCV genome or transient expression of its core, Е1/Е2, NS3 and NS5B led to the death of 20–47% cells, 72 h posttransfection, whereas the expression of the NS4A/B, NS5A or NS3-NS5B polyprotein did not affect cell viability. HCV proteins caused different impacts on the activation of caspases-3, -8 and -9 and on DNA fragmentation. The structural core and E1/E2 proteins promoted apoptosis, whereas non-structural NS4A/B, NS5A, NS5B suppressed apoptosis by blocking various members of the caspase cascade. The majority of HCV proteins also enhanced autophagy, while NS5A also induced necrosis. As a result, the death of Huh7.5 cells expressing the HCV core was induced via apoptosis, the cells expressing NS3 and NS5B via autophagy-associated death, and the cells expressing E1/E2 glycoproteins or harboring HCV the replicon via both apoptosis and autophagy.

## 1. Introduction

The hepatitis C virus (HCV) is a wide-spread etiological agent that often leads to chronic liver disease. Chronic hepatitis C (CHC) is strongly associated with the development of liver fibrosis, cirrhosis, and hepatocellular carcinoma (HCC). The exact mechanisms of HCV pathogenesis are still not completely understood. Numerous evidence suggest that pathogenic events are promoted by hepatocyte death in various forms such as apoptosis, autophagy, and necrosis [1,2,3]. Apoptosis plays a prominent role in maintaining tissue homeostasis by the removal of senescent cells which have been damaged by various factors including viral infections, or by the removal of rapidly proliferating transformed cells. The outer apoptotic pathway is mediated by the transmembrane death receptors that belong to the tumor necrosis factor (TNF) family and by activation of the initiator caspase-8 [4]. The inner pathway is induced in response to DNA damage, pronounced oxidative stress, increase of intracellular Са^2+^ levels, ER stress and unfolded protein response (UPR) [4]. These events lead to mitochondrial dysfunction that triggers activation of the initiation caspase-9 [4,5]. Both outer and inner pathways activate the key effector caspase-3 [4]. The latter, in its turn, triggers the breakdown of nucleus-activating DNAases that degrade DNA to nucleosome fragments. HCV infection was earlier shown to induce both of these caspase-mediated apoptotic pathways. In particular, HCV directly triggers unfolded protein response and calcium-dependent mitochondrial dysfunction [6]. Normally, apoptotic cell death is not accompanied by inflammation. However, persistent viral infections and extensive production of pro-apoptotic stimulus (death receptors, inner signals such as cytochrome c, Bax, Bad and Bak proteins) promote intensive and long-lasting inflammation that can result in massive loss of hepatocytes, ischemic liver damage, and irreversible organ damage to follow [7]. At the same time, several HCV proteins were shown to suppress apoptosis, thus preventing the elimination of HCV-infected cells, and promoting virus persistence [2]. Dysregulation of proapoptotic pathway,s such as the disruption of Bcl-2 and Bcl-xL proteins, facilitates cell proliferation, possibly contributing to hepatocarcinogenesis [8]. Various HCV-encoded proteins play different roles in modulating apoptosis by interacting and interfering with host factors, but literature data on the role of individual HCV proteins in modulation of apoptosis are controversial [2,8,9,10,11].

HCV also promotes autophagy, as revealed both in vitro in various hepatoma cell lines and in vivo by analyzing biopsies from CHC patients [1,2,3,12]. Autophagy represents a conservative regulated mechanism during which cell organelles are targeted for degradation in autolysosomes in order to preserve cell homeostasis. Autophagy is generally regarded as a pro-survival event. However, intensive formation of autophagosomes, enhanced permeabilization of mitochondrial membrane and critical decreases in ATP levels can trigger an “autophagy-associated cell death” [4] similar to that observed during acute HCV infection [1,13]. Interplay between autophagy and HCV infection remains controversial. On one hand, Sir et al. revealed a co-localization of the main autophagy marker LC3 (microtubule associated protein 1 light chain 3) with HCV replicase proteins on autophagosome membranes [14], and it was shown that inhibitors of autophagy suppress HCV replication in cell culture [15,16]. One the other hand, Mohl et al. demonstrated that at least NS5A, one of HCV replicase components, does not co-localize with autophagosomes during active replication of the virus [17]. Several authors have proposed that HCV can initiate the formation of autophagosomes, whereas this process can be blocked at later stages [18]. In support, HCV-induced autophagy has been assumed to alter innate immune response and facilitate cell proliferation by blocking apoptosis [19,20]. At the same time, others observed that HCV infection induced autophagy accompanied by lysosomal activity [2].

It is assumed that a high viral load during HCV infection promotes hepatocyte death through necrosis (or necroptosis, its regulated form), whereas low viral load can induce apoptosis [21,22]. Necrosis represents another type of cell death [4]. It is mediated by acute tissue injury, and is characterized by the loss of cell membrane integrity with the concomitant release of metabolites into the extracellular space. Necrosis triggers both a toxic response in the surrounding live cells and an immune response. However, there are still only scarce data about the mechanisms of how the virus triggers necrosis, and on the virus proteins that mediate this response.

All the above-mentioned information shows that exact pathways which convey HCV-induced metabolic and stress responses still remain questionable. There are debates about virus regulation of the hepatocyte proliferation/death, and the role of individual proteins of the virus remains obscure.

The goal of this study was to estimate the impact of HCV proteins on autophagy, apoptosis, and necrosis in the human hepatoma Huh7.5 cell line by analyzing the respective markers in cells transiently expressing the core, E1/E2, NS3, NS4A/B, NS5A, and NS5B proteins individually or simultaneously, as well as in cells harboring the HCV full-length replicon, and thus, expressing the whole proteome at a higher physiological level.

## 2. Results

### 2.1. Several HCV Proteins Hamper Huh7.5 Cell Growth

First, an influence of HCV proteins on growth of Huh7.5 cells was analyzed. The cells were transfected with the plasmids encoding individual proteins or with the NS3-NS5B polyprotein, were stained with 4′,6-diamidino-2-phenylindole (DAPI) 72 h posttransfection, and their quantities were estimated using a fluorescent microscope. Transfection efficacy was estimated by immunofluorescent staining of the HCV protein in cells with monoclonal antibodies. The images are presented in the Figure S1. In all cases, the efficacy exceeded 60% with no statistical differences between the plasmids. Moreover, no pronounced difference was found in the intensity of fluorescence between the cells’ expression of the individual HCV proteins, NS3-NS5B polyprotein, or cells harboring the replicon. Analysis of the expression of NS3, NS5A, and NS5B proteins in cells expressing these proteins and the ones with the replicon was performed by western blot analysis. It revealed that in the case of the HCV replicon, the levels of NS5B were lower than in case of their overexpression (Figure S2).

**Figure 1:**
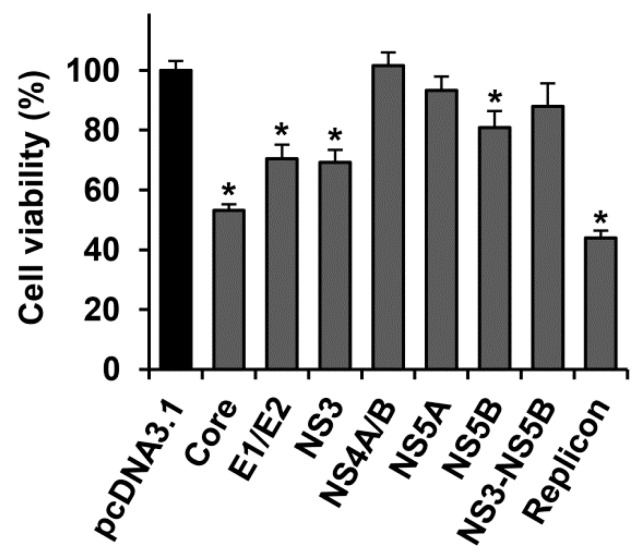
Several hepatitis C virus (HCV) proteins hamper growth/viability of Huh7.5 cells. The cells, transfected with plasmids encoding HCV proteins, were counted 72 h posttransfection by microscopy visualization, and the values were normalized to the number of cells transfected with the empty pcDNA3.1(+) vector. The data are presented as means ± standard error of the mean (SEM) of eight measurements done in three independent experiments. * *p* < 0.05 vs. cells transfected with pcDNA3.1(+) vector (black bar).

The expression of NS4A/B and NS5A, as well as of NS3-NS5B polyprotein did not affect cell growth and viability, compared to Huh7.5 cells transfected with the empty pcDNA3.1(+) vector (Figure 1). In its turn, transfection with this vector did not alter the viability of cells, as compared to the untransfected Huh7.5 cells. NS5B caused a minor (<20%) decrease in the number of living cells, whereas E1/E2 glycoproteins and NS3 protein caused a more pronounced decrease in the number of living cells (~30%). The highest decrease in the number of cells (47–56%) was observed in case of the transient expression of the HCV core, and stable replication of the full-length genome of the virus (the HCV replicon).

### 2.2. HCV Proteins Exhibit Different Regulatory Activity towards Apoptotic Pathways

**Figure 2:**
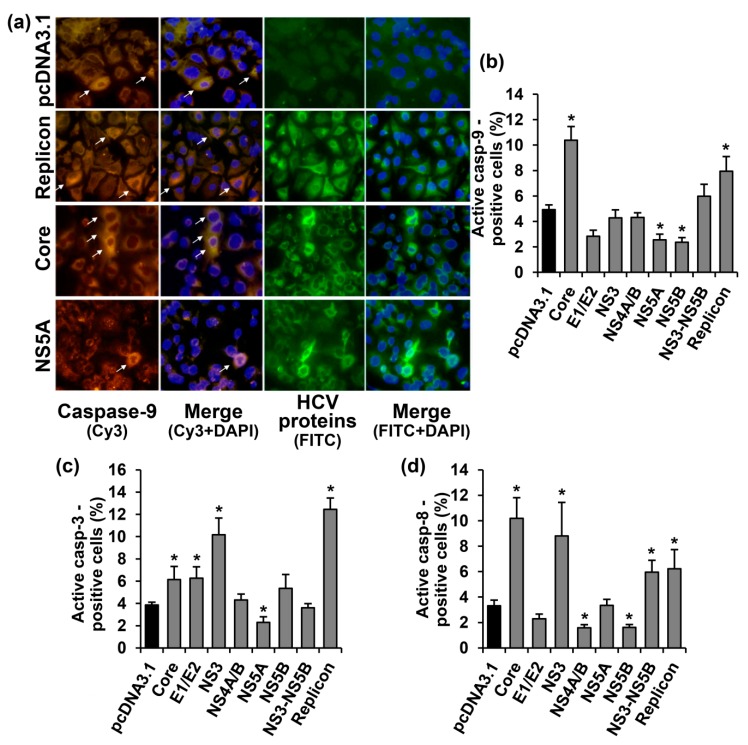
HCV proteins affect activation of caspases-3, -8 and -9 in Huh7.5 cells in different manners. (**a**) Immunofluorescent staining of the activated caspase-9 and HCV proteins in Huh7.5 cells transiently expressing the HCV core or NS5A proteins, or harboring the full-length HCV replicon (400× magnification). Vertical panels left to right: staining with rabbit anti-caspase-9 primary and anti-rabbit secondary antibodies conjugated to Cy3 (orange), merge with nuclear staining with DAPI (blue), staining with mouse monoclonal antibodies to HCV proteins and anti-mouse secondary antibodies conjugated to fluoresceine isothiocianate (FITC; green), combined with nuclear staining with DAPI (blue). The arrows indicate caspase-9 positive cells. (**b**–**d**) Percentages of the cells which tested positive for the caspases-9 (**b**), -3 (**c**), and -8 (**d**). Values on each diagram are means ± SEM of eight measurements done in three independent experiments, * *p* < 0.05 compared to the cells transfected with the empty vector (black bar).

Our next step was to investigate possible mechanisms of the apoptosis induction during the expression of HCV proteins. The induction of apoptosis was accessed by quantifying activated caspases-3, -8, and -9 that mediate major apoptotic pathways. These activated caspases were detected in the cytoplasm of the cells, using the specific antibodies, as homogenous intensive staining. Typical images, exemplified in caspase-9, are presented in Figure 2a, and the quantification of the data for all three caspases is given in the Figure 2b–d. Different caspases were present in the cells with different rates of detection, depending on the HCV protein expressed.

Caspase-9 was detected in 4.9% cells transfected with the empty vector control. Expression of HCV NS5A and NS5B proteins reduced the number of the caspase-positive cells by two-fold, whereas the core protein increased the number of cells with the activated caspase-9 by an additional 2.1-fold, compared to the vector (Figure 2a,b). Expression of other HCV proteins, as well as of NS3-NS5B polyprotein, had no statistically significant effect. Finally, Huh7 cells harboring the HCV replicon exhibited a 1.6-fold increase in the number of cells with the activated caspase, compared to the control cells.

Activation of caspase-3 was detected in 3.9% Huh7.5 cells transfected with the empty vector (Figure 2c). NS5A protein reduced the number of the cells with the activated caspase-3, whereas core, E1/E2, and NS3 proteins increased the rate of detection of the activated caspase by 1.6–2.6-fold. A similar increase (3.2-fold) was also observed in cells harboring the full-length HCV replicon.

**Figure 3:**
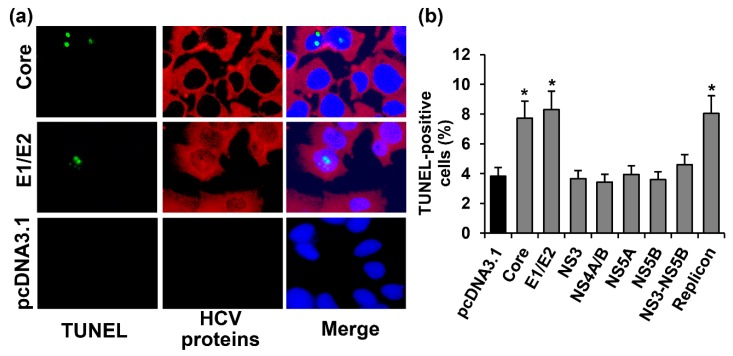
The HCV core and E1/E2 increase the number of Huh7.5 cells with nuclear DNA fragmentation, i.e., at the end stage of apoptosis. (**a**) Huh7.5 cells transfected with the core- and E1/E2-expressing plasmid or the empty pcDNA3.1 vector were stained 72 h posttransfection with the “DeadEnd™ Fluorometric terminal deoxynucleotidyl transferase dUTP nick end labeling (TUNEL) System” kit (green), with mouse monoclonal antibodies on HCV proteins and anti-mouse secondary antibodies conjugated to Alexa Fluor 594 (AF594), and with DAPI. Vertical panels left to right: TUNEL staining (green), HCV proteins (red), and overlay of TUNEL, HCV proteins and DAPI staining; (**b**) Percentages of TUNEL-positive cells. Values are means ± SEM of eight measurements done in three independent experiments, * *p* < 0.05 compared to the cells transfected with the empty vector (black bar).

Activated caspase-8 was detected in 3.3% cells transfected with the empty vector (Figure 3d). Expressions of NS4A/B and NS5B proteins led to a decrease in the number of caspase-8 positive cells by two-fold, whereas the HCV core, NS3, NS3-NS5B polyprotein and the virus replicon increased the number of such cells by 3.1, 2.7, 1.8, and 1.8-fold, respectively, compared to the vector.

DNA fragmentation, the end stage of apoptosis, was studied by the terminal deoxynucleotidyl transferase dUTP nick end labeling (TUNEL) approach. Such staining of cells allowed the visualization of a bright fluorescent signal in the nuclei of some cells (Figure 3a). In the Huh7.5 cells transfected with the empty vector, 3.8% of the population exhibited signs of DNA fragmentation (Figure 3b). The increase in the number of TUNEL-positive cells was observed for the cells expressing the core and Е1/Е2 proteins, or harboring the HCV replicon. In the case of other HCV proteins, no statistically significant changes were found.

### 2.3. Several HCV Proteins Stimulate Autophagy in Huh7.5 Cells

**Figure 4:**
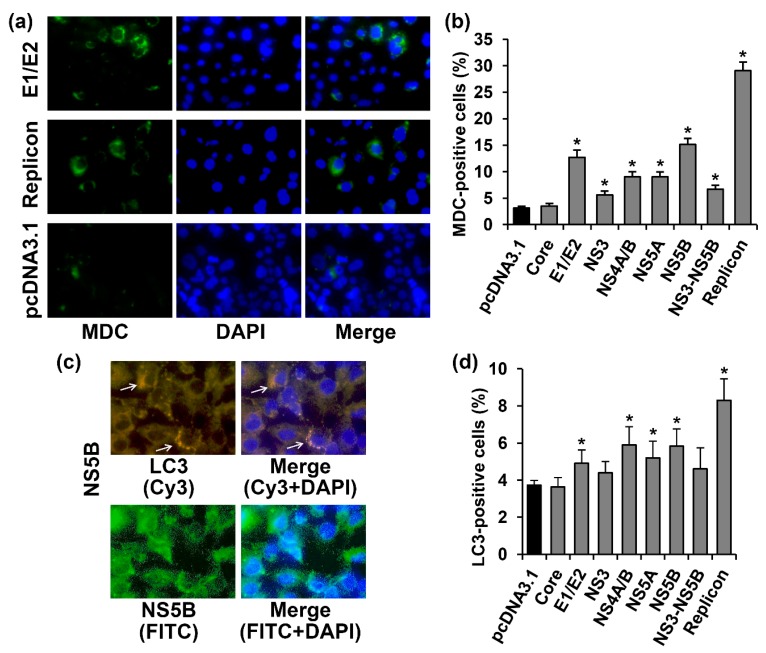
HCV proteins activated autophagy in Huh7.5 cells, as revealed by the enhanced incorporation of monodansylcadaverine into autophagosomes and detection of LC3. (**a**) Huh7.5 cells harboring the HCV replicon, or transfected with the E1/E2-expressing plasmid or the empty pcDNA3.1(+) vector were stained 72 h posttransfection with the monodansylcadaverine (MDC) and with DAPI. Vertical panels from the left to the right are: MDC staining (green), nuclear staining with DAPI (blue), overlay of MDC and DAPI staining; (**b**) Percentages of MDC-positive cells. (**c**) immunofluorescent staining of the activated LC3 and NS5B protein in Huh7.5 cells (400× magnification). Vertical panels left to right: staining with rabbit anti-LC3 primary and anti-rabbit secondary antibodies conjugated to Cy3 (orange) or primary mouse anti-NS5B antibody and secondary anti-mouse antibodies conjugated to FITC (green), merge with nuclear staining with DAPI (blue) The arrows indicate cells with LC3 punctuate staining; (**d**) Percentages of LC3-positive cells. Values are means ± SEM of eight measurements done in three independent experiments, * *p* < 0.05 compared to the cells transfected with the empty vector (black bar).

Autophagy was assessed by visualizing the incorporation of monodansylcadaverine (MDC) into the living cells using an appropriate kit from Abcam. The cells with activated autophagy were identified as cells with a bright fluorescent signal in the cytoplasm (Figure 4a). Among the Huh7.5 cells transfected with the pcDNA3.1(+) vector, 3.1% of the cells exhibited signs of autophagy (Figure 4b). The expression of most of the studied HCV proteins (E1/E2, NS3, NS4A/B, NS5A, and NS5B, as well as the NS3-NS5B polyprotein) independently or in the context of the full-length replicon led to a 1.8–9.3-fold increase of MDC incorporation. The only virus protein that did not affect the incidence of autophagy was the HCV core. To verify this result, the cells transfected either with the vector or with the core-expressing plasmid were incubated with 60 µM chloroquine, an autophagy inhibitor, or with 250 nM rapamycin, a standard autophagy inducer, for 3 h. Indeed, chloroquine suppressed MDC incorporation in both cases by 2.1–2.7-fold. In contrast, rapamycin augmented the autophagy incidence by 1.8–2.3-fold. Thus, the HCV core neither provoked autophagy nor inhibited it, if induced by other stimuli.

Additional verification of these results was performed by the staining of microtubule-associated protein 1A/1B-light chain 3 (LC3). During the formation of autophagosomes, LC3 protein is conjugated to phosphatidylethanolamine, leading to the accumulation of this product and an increase in the level of fluorescence. In our experiments, we observed an increase in the number of cells with enhanced fluorescence in punctuate structures in the cytoplasm in the case of the expression of HCV proteins (as is exemplified in Figure 4c). Among Huh7.5 cells transfected with the empty vector, only 3.7% tested positive for LC3. The expression of E1/E2, NS4A/B, NS5A, and NS5B proteins led to the 1.3–1.6-fold increase in LC3–positive cells (Figure 4d). In the case of cells harboring the HCV replicon, >8% of cells demonstrated bright fluorescence. The HCV core had no effect on the number of cells with strong LC3 staining, whereas the expression of NS3 or NS3-NS5B resulted in a tendency of the polyprotein to increase (*p* = 0.15 and 0.09, respectively). Thus, a comparison of the LC3 staining and MDC accumulation revealed similar results, with LC3 staining being less sensitive.

### 2.4. HCV NS5A Protein Moderately Increases the Incidence Rate of Necrosis

**Figure 5:**
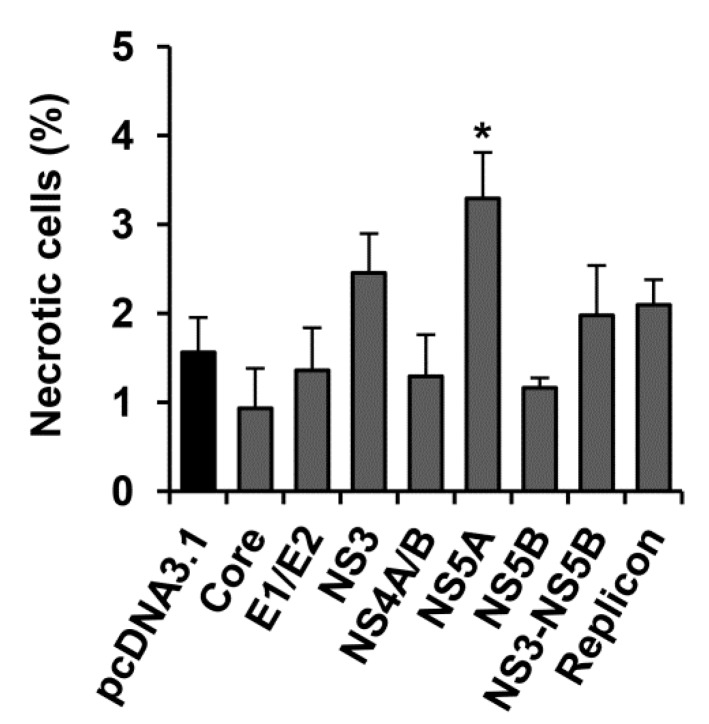
HCV NS5A protein promotes necrotic cell death. Huh7.5 cells harboring the HCV replicon, or transfected with the HCV-expressing plasmid or the empty pcDNA3.1(+) vector were stained 72 h posttransfection with trypan blue, and the stained cells were counted. The bars represent percentages of trypan-positive cells. Values are means ± SEM from three independent experiments, * *p* < 0.05 compared to the cells transfected with the empty vector (black bar).

Necrotic cell death was evaluated by determining damage to the plasma membrane. Among the control Huh7.5 cells transfected with the pcDNA3.1(+) vector, not more that 1.6% demonstrated signs of necrosis. A statistically significant 2.1-fold increase in the number of necrotic cells was observed only in the case of the expression of the HCV NS5A protein: in this case 3.3% necrotic cells were detected (Figure 5).

## 3. Discussion

In the current study we present an analysis of the influence of expression of various structural and non-structural HCV proteins on the viability/proliferation of hepatoma Huh7.5 cells. It is a well-established fact that chronic hepatitis C is accompanied by the continuous death of infected hepatocytes. It has been shown in the context of both the liver of chronic hepatitis C patients and in vitro in the infected primary or immortalized hepatocytes [10,21]. However, the input of individual proteins of the virus in the modulation of cell fate still remains disputable. In order to investigate mechanisms of cell death in response to the expression of HCV proteins, we used an array of immunocytochemical approaches for the detection of five markers of apoptosis and autophagy, as well as a cytological approach for necrosis detection. The results are summarized in Table 1.

The results suggest that the overexpression of core and E1/E2 proteins as well as the continuous replication of the virus genome RNA trigger apoptosis (Table 1). The core protein induced the activation of initiator caspases-8 and -9, with subsequent activation of the effector caspase-3 that triggered DNA fragmentation and cell death. The involvement of the HCV core in the receptor-mediated apoptosis is in agreement with our previous findings that this protein triggers the secretion of tumor necrosis factor α (TNF-α) when expressed in Huh7.5 cells [23]. Moreover, the data of the current study confirm several reports from other groups about the pro-apoptotic effect of the core protein [8,24,25]. In addition to cell systems, induction of apoptosis by the HCV core was also described in vivo in transgenic mice [26,27,28]. Core-mediated apoptosis can resulte from the activation of the mitochondrial signaling pathway involving activation of the Bax (*B*cl-2-like protein 4) protein via the disruption of calcium homeostasis and concomitant mitochondria dysfunction, as well as from the TNF-dependent pathway [2,24,29]. However, the HCV core was also reported to block apoptosis by various mechanisms thus contributing to core-driven hepatocarcinogenesis [8,30,31]. The discrepancy of these data could be explained by a “competitive model” of the adaptive cell response to the stress, meaning that both lethal and pro-survival programs took place with the overall effect depending on variability in experimental settings in vitro [32]. It could be speculated that both events could take place in the liver: dying hepatocytes could contribute to the development of inflammation and liver damage, whereas survival of the stressed infected cells predisposes them to the malignant transformation.

We observed that the surface E1/E2 glycoproteins increased the incidence of the activated caspase-3 and concomitant apoptotic cell death (Table 1). At the same time, we failed to detect any statistically significant enhancement in the activation of initiator caspases-8 and -9. Further studies of the mechanisms of E1/E2-mediated cell death should be considered. The data on the involvement of these proteins in apoptosis is scarce. However, most authors conclude that E1/E2 pro-apoptotic actions during their expression in various cell systems takes place due to both outer TNF-dependent [33] and inner mitochondrial pathways [33,34,35]. Since we previously observed the enhanced TNF-α production in E1/E2-overexpressing Huh7.5 cells, apoptosis is likely to be mediated by an outer pathway [23]. Therefore, both glycoproteins may trigger apoptosis via other signaling cascades. It may be assumed that apoptosis in such cells can be triggered by the activation of caspase-4, which does not affect the status of caspase-3 [36], or via caspase-12, which activates both caspase-3 and -9 [37]. These caspases can be activated by ER stress and unfolded protein response [36,37] that accompany the expression of E1 and E2 glycoproteins [38]. However, this assumption merits further studies.

The NS3 protein was shown to trigger the activation of caspase-3 via the activation of caspase-8 through the outer apoptotic pathway (Table 1). This is in agreement with our previous findings of the enhanced accumulation of TNF-α in the cells overexpressing NS3 protease [23]. However, this process does not lead to the accomplishment of apoptosis, since no DNA fragmentation was observed. Activation of the caspase-8 in the presence of the NS3 protein was already reported [39]. Epithelial cells and fibroblasts, exposed to the recombinant NS3 protein, exhibited enhanced production of pro-inflammatory cytokines, augmented reactive oxygen species (ROS) production thus leading to apoptosis [25]. In contrast, NS3 was also shown to demonstrate anti-apoptotic activity that was realized through interaction with tumor suppressor p53 [40].

Our data indicate that the individual non-structural proteins NS4A/B, NS5A and NS5B suppressed various stages of activation of the caspase cascade (Table 1). NS5A prevented the activation of the caspase-3, presumably through the inhibition of caspase-9. Notably, this is in agreement with the literature data, as this protein was shown earlier to use multiple strategies to block inner pathways of apoptosis by interfering with various host factors [8,9,41]. Since the inner pathway of apoptosis is activated through mitochondrial dysfunction, the reason why NS5A did not induce it while the HCV core did does not seem obvious; both of them triggered the massive production of ROS [42,43]. It can be speculated that such difference is due to the different actions of these proteins on the ROS-producing systems of the cell. We have recently demonstrated that enhanced productionof reactive oxygen species (ROS) in NS5A-expressing cells does not result from the efflux of calcium ions from endoplasmic reticulum (ER) and their accumulation in mitochondria [44] that occur in the case of the HCV core [29]. However, other possible mechanisms accounting for these events cannot be excluded.

The NS5B protein exhibited a moderate suppressive effect towards the initiation of caspases-8 and -9 (Table 1), thus inhibiting apoptosis. There are only scarce data about the role of this protein in the development of apoptosis. The pro-apoptotic activity of NS5B was assigned to its ability to interact with the pro-apoptotic BIK protein [11], whereas its anti-apoptotic activity was assigned to its ability to suppress NF-κB-dependent anti-apoptotic Bcl-xL and XIAP proteins during treatment with TNF-α [45]. Such a discrepancy could result from the usage of different cell models and expression vectors.

Huh7.5 cells expressing NS4A/B showed the suppression of caspase-8 activation while other apoptotic markers remained unaffected (Table 1). The literature reports that individual NS4A and NS4B proteins can either stimulate or block apoptosis, depending on the cell model and the expression status (transient or stable expression) [10,45,46]. In our experiments, NS4A was expressed together with NS4B, and we failed to find any data on the effect of such combination on cell death.

Another aspect of our study was an investigation of the NS3-NS5B polyprotein effect on cell death. Interestingly, a combination of the non-structural proteins activated caspase-8 with no effect on DNA fragmentation, which takes place during apoptosis (Table 1). Since individual HCV proteins exhibited different actions on apoptosis, it could be assumed that the pro-apoptotic activity of NS3 may be balanced by the anti-apoptotic actions of NS4A/B, NS5A, and NS5B. In addition, the expression of NS3 and NS5A proteins, along with other virus proteins, changes their localization, in contrast with the overexpression of the individual proteins [46,47]. Finally, in similar conditions, Lim et al. reported the activation of caspase-3 in response to the expression of NS3-NS5B polyprotein in primary human hepatocytes (PHH) but not in hepatoma Huh7 cells, suggesting that Huh7.5, being tumor-derived cells, are more resistant to pro-apoptotic stimuli [21].

Our study revealed that the expression of HCV proteins in the full-length replicon leads to the activation of both the apoptotic pathways. Previously it was reported that Huh7.5 cells harboring the subgenomic replicon or infected by recombinant variants of the virus in the HCV cell culture (HCVcc) system activated FAS- and TRAIL-mediated outer and inner pathways of apoptosis through the induction of the unfolded protein response, pro-apoptotic Bax protein and cytochrome c release [2,46,48]. As the same was observed only for the core protein, this protein has the most pronounced impact on apoptosis induction in HCV-infected cells.

Autophagy in the living cells was analyzed by measuring the accumulation of fluorescent monodansylcadaverine in autolysosomes that are formed during late stages of autophagy when autophagosomes fuse with lysosomes. Moreover, MDC is co-localized with the main marker of autophagosomes—the LC3 protein which can be detected during all stages of autophagosome formation [49,50]. Enhanced autophagy in Huh7.5 cells in response to the expression of E1/E2, NS4A/B, NS5A, NS5B proteins individually or in the context of the full-length replicon was revealed by two independent approaches (Table 1). Differences were found in the case of NS3 protein and NS3-NS5B polyprotein, for which only a tendency to statistically significant accumulation of LC3 was observed. This could be due to a lower sensitivity of this approach, compared to the visualization of MDC accumulation, and due to the fact that these autophagy markers are different in kind. In general, these data are in agreement with previous findings of the other groups that showed enhancement of autophagy by the hepatitis C virus [1,2,3,31,51]. For example, Chu et al. also reported that the induction of autophagy is achieved independently of HCV core expression, but that it is dependent on nonstructural proteins [51]. The difference between our data and literature data is with regards to the impact of individual proteins of the virus on this process. We show that the formation of autophagosomes is promoted mostly by NS5B and Е1/Е2 proteins, whereas other groups reported activation of autophagy by NS5A [18,52], NS4B [53], the core, NS3/4A and NS4B [54].

Though analysis of apoptosis and autophagy was performed by the quantification of biochemical markers, necrotic cells with altered plasma membranes were visually detected. The increased number of necrotic cells was detected only when the NS5A protein was expressed (Table 1). Notably, NS5A exhibited no statistically significant effect on the incidence of necrosis when expressed with other nonstructural proteins (i.e., in cells transfected with the plasmid encoding NS3-NS5B, or harboring the HCV replicon). This could be due to several reasons including different expression levels of NS5A, or its different localization. However, a comparison by Western blotting between accumulation levels of NS5A in cells transfected with pcNS5A, and those harboring the HCV replicon revealed no significant differences (Figure S2). Thus, NS5A may have a different distribution between organelles when expressed individually or together with other HCV proteins. Nevertheless, our results indicate that at least a part of the NS5A-expressing cells dies by necrosis. An impact of necrosis on the death of the HCV-infected hepatocytes is still not fully understood. To our knowledge, there are no data showing the promotion of necrosis by the hepatitis C virus and its proteins. The only exception is the data of Lim et al., who demonstrated the occurrence of necroptosis (i.e., a programmed necrosis) in the cells expressing HCV proteins, and its prevention by its inhibitor necrostatin-1 [21]. Interestingly, in HCV-unrelated studies, caspase-8 was shown to inhibit necrosis [55]. Nevertheless, it is worth noting that in our study, necrosis was detected only in NS5A-expressing cells where no activation of caspase-8 was observed (Table 1).

We assume that different effects of HCV proteins on the induction of apoptosis, necrosis, and autophagy do not result from different levels of their expression. For example, the activation of caspases and DNA fragmentation was more pronounced for the core protein than for NS5A and NS5B proteins. Previously we quantified the expression of these proteins in Huh7 cells and revealed that HCV the core is accumulated up to 100 fg/cell, whereas NS5A and NS5B reached levels of 700 fg/cell each [43]. The same levels of these nonstructural proteins also do not correlate with their different effects on necrosis. Finally, the cells harboring the replicon exhibited higher rate of apoptotic cell death than NS5B-expressing cells despite lower levels of its expression (Figure S2).

The presented results show that HCV proteins expressed alone or in cells harboring the replicon promote cell death by several pathways. At the same time, Pietschmann et al. established that the HCV subgenomic replicon can propagate for up to 6 months without complete loss in Huh7 cells, even in the absence of G418 [56]. Our data do not contradict that paper. First, a decrease in viral RNA levels during cell passaging showed a moderate negative effect of the virus on the cells. Second, in our case we used the full-length HCV replicon (which encoded the core protein) compared to the subgenomic replicon of that study. It is accepted that the HCV core is more hazardous for Huh7 cells among all the proteins of the virus. Third, in our hands, less than 10% of cells exhibited activation of caspases, signs of pronounced DNA fragmentation, or of necrosis. Thus, in vitro it correlates with slower the cell growth evidenced by many groups. In the context of infection, it also falls in line with an increased incidence of hepatocyte death [57].

## 4. Materials and Methods

### 4.1. Cells

The naive Huh7.5 cell line [58] was cultured in Dulbecco’s modified minimal essential medium (DMEM) (Paneco, Moscow, Russia) supplemented with 10% fetal calf serum (Gibco, Waltham, MA, USA), 2 mM glutamine, 50 µg/mL gentamycin at 37 °C in a humid atmosphere with 5% CO_2_ · Huh7 cells harboring a bicistronic full-length I_389_/core-3′/5.1 HCV replicon [59] were cultured similarly in DMEM/F12 medium (Paneco) containing fetal calf serum, glutamine and supplemented with 300 µg/mL geneticin (G418, Invitrogen, Waltham, MA, USA).

### 4.2. Plasmids

The plasmids encoding individual HCV cores (pcCore), E1 and E2 (pcE1E2), NS3 (pcNS3), NS4A and NS4B (pcNS4), NS5A (pcNS5A) and NS5B (pcNS5B) were described previously [43,60,61,62]. In addition, a plasmid pcNS3-NS5B encoding NS3, NS4, NS5A and NS5B as a single polyprotein was used [61]. All these plasmids were constructed using pcDNA3.1(+) vector (Invitrogen). The plasmids were propagated in XL-1 blue *Escherichia coli* strain and purified using QIAGEN^®^ Plasmid Purification Maxi Kit (Qiagen Inc., Hilden, Germany).

### 4.3. Transfection

Twenty four hours prior to transfection Huh7.5 cells were seeded in a 24-well plate (SPL Lifesciences, Pocheon, Korea) with a microscope cover glass at a density of 5 × 10^4^ cells/well. The transfections were carried out using TurboFect Transfection Reagent (Thermo Fisher Scientific, Rockford, IL, USA) according to manufacturer’s specification. Seventy two hours posttransfection the cells were washed with PBS and either studied directly or fixed as described below.

### 4.4. Immunocytochemical Analysis

HCV proteins and markers of cell death in the transfected cells were detected by immunocytochemical analysis. For detection of apoptosis and autophagy primary rabbit antibodies to activated caspases-3, -8, -9, and LC3A/B (ab52293, ab4052, ab32539, and ab128025, respectively, Abcam, Cambridge, UK), and secondary Cyanine 3.29-OSu (Cy3)—conjugated antibodies to anti-rabbit IgGs (ab6939, Abcam) were used. For this analysis the cells were fixed by incubation with methanol for 20 min at –20 °С. Autophagy was detected also in viable cells using the “Autophagy Detection Kit” (ab139484, Abcam) according to the manufacturer's protocol. Nuclei were stained with 4′-6-diamino-2-phenylindole dye (DAPI).

DNA fragmentation was analyzed in paraformaldehyde-fixed cells by TUNEL assay using “DeadEnd™ Fluorometric TUNEL System” kit (G3250, Promega, Madison, WI, USA) according to vendor’s instructions.

HCV proteins were detected by indirect staining using monoclonal antibodies to the core (1F9), NS3 (2Н4), NS4 (a mixture of 3F12 to NS4A and 6B11 to NS4B proteins), and NS5A (3F4) proteins, raised and described by us previously [63], as well as by commercially-available monoclonal antibodies to E1 and E2 glycoproteins (using a mixture of ab21306 and ab20852, Abcam), or to the NS5B protein (sc-58146, Santa Cruz Biotechnology, Dallas, TX, USA). As secondary antibodies, fluoresceine isothiocianate-labeled anti-mouse IgGs (ab97039, Abcam) were used. In the DNA fragmentation assay HCV proteins were stained using the primary antibodies listed above and secondary Alexa Fluor 594 (AF594)-labeled anti-mouse IgGs (R37121, Thermo Fisher Scientific).

Simultaneous detection of HCV proteins and markers of apoptosis (caspases-3, -8, and -9) was performed by double immunocytochemical staining with mixtures of the respective antibodies and conjugates.

Staining was visualized by the fluorescent microscope Axio Scope A1 Carl Zeiss (Germany) at excitation/emission wavelengths of 520/560 nm (FITC), 552/565 nm (Cy3), 590/617 nm (AF594), and 360/460 nm (DAPI). The stained cells were counted in no less than 8 different fields of view at 400× magnification by two independent observers.

### 4.5. Necrosis

Necrosis was accessed using a widely used trypan blue assay that stains the cells with altered membrane permeability [64,65]. Briefly, the cells were treated with trypan blue and the stained cells were counted on a microscope using a Goryaev chamber.

### 4.6. Immunoblot Analysis

The Western blot analysis was performed as described previously [43]. The primary antibodies were used in 1% (*w*/*v*) non-fat milk in the following concentrations: anti-actin (Abcam, ab3280, 0.2 µg/mL), anti-NS3 (2H4, 1 µg/mL), anti-NS5A (3F4, 1.8 µg/mL), anti-NS5B (Santa-Cruz, sc-58146, 0.5 µg/mL). The HRP-conjugated anti-mouse (Santa-Cruz, sc-2005) secondary antibodies were used at a concentration of 0.07 µg/mL. The signal was visualized using Pierce ECL Western Blotting substrate (Thermo Scientific, Waltham, MA, USA), with the exception of NS5B that was visualized using SuperSignal West Femto Maximum Sensitivity Substrate (Thermo Scientific).

### 4.7. Statistical Analysis

Statistical analysis was performed with “Statistica 6” software (StatSoft Inc., Tulsa, OK, USA). All data on the graphs were presented as mean ratios of the number of stained cells to the total number of cells ± standard error of means. The significance of differences between two groups were determined using a Student’s *t-*test. A *p*-value < 0.05 was considered statistically significant.

## 5. Conclusions

In conclusion, the data presented in this study point to different mechanisms by which HCV proteins modulate cell death. For the first time, markers of apoptosis, necrosis, and autophagy were analyzed simultaneously in hepatoma cells overexpressing individual virus proteins or their combinations, or harboring the full-length replicon to ensure their more physiological expression in the context of HCV genome replication. Most HCV proteins were shown to promote autophagy and to exhibit different actions of apoptotic pathways. The structural core and E1/E2 proteins contributed to apoptotic cell death, whereas the non-structural NS4A/B, NS5A, and NS5B proteins suppressed it by blocking various members of the caspase cascade. A comparison of the viability of the transfected cells with rates of detection of cell death markers suggests that death of NS3- and NS5B-expressing cells occurs as a result of autophagy, and death of core-expressing cells occurs through the apoptotic program, whereas E1/E2 glycoproteins favor both apoptotic and autophagy cell death. Finally, a stable cell line harboring the full-length replicon exhibited strong signs of both apoptosis and autophagy. Our findings suggest possible molecular and cellular mechanisms that may present a target for the treatment and/or prevention of virus-associated pathologies.

## Figures and Tables

**Table 1:** Impact of HCV proteins on promoting various types of death of Huh7.5 hepatoma cells.

Markers of Cell Death		HCV Proteins							
		Core	E1/E2	NS3	NS4A/B	NS5A	NS5B	NS3-NS5B	HCV Replicon
Apoptosis	Caspase-8	3.1 ^1^	~ ^2^	2.7	0.5	~	0.5	1.8	1.9
	Caspase-9	2.1	~	~	~	0.5	0.5	~	1.6
	Caspase-3	1.6	1.6	2.6	~	0.6	~	~	3.2
	TUNEL	2.0	2.2	~	~	~	~	~	2.1
Auto-phagy	MDC	~	4.1	1.8	2.9	2.9	4.8	2.1	9.3
	LC3-II	~	1.3	~	1.6	1.4	1.6	~	2.2
Necrosis		~	~	~	~	2.1	~	~	~

^1^ Changes in rates of detection of the respective marker (fold). ^2^ Symbol “~” denotes absence of a statistically significant effect.

## Supplementary Materials

The following are available online at www.mdpi.com/1422-0067/18/11/2346/s1.
