# Supplementary material for: Modulation of Cell Death Pathways by Hepatitis C Virus Proteins in Huh7.5 Hepatoma Cells
Source: Int J Mol Sci. 2017 Nov 6;18(11):2346. doi: 10.3390/ijms18112346 (PMC5713315; doi:10.3390/ijms18112346)
Supplement: Supplementary file 1 [file ijms-18-02346-s001.pdf]

SUPPLEMENTARY MATERIAL

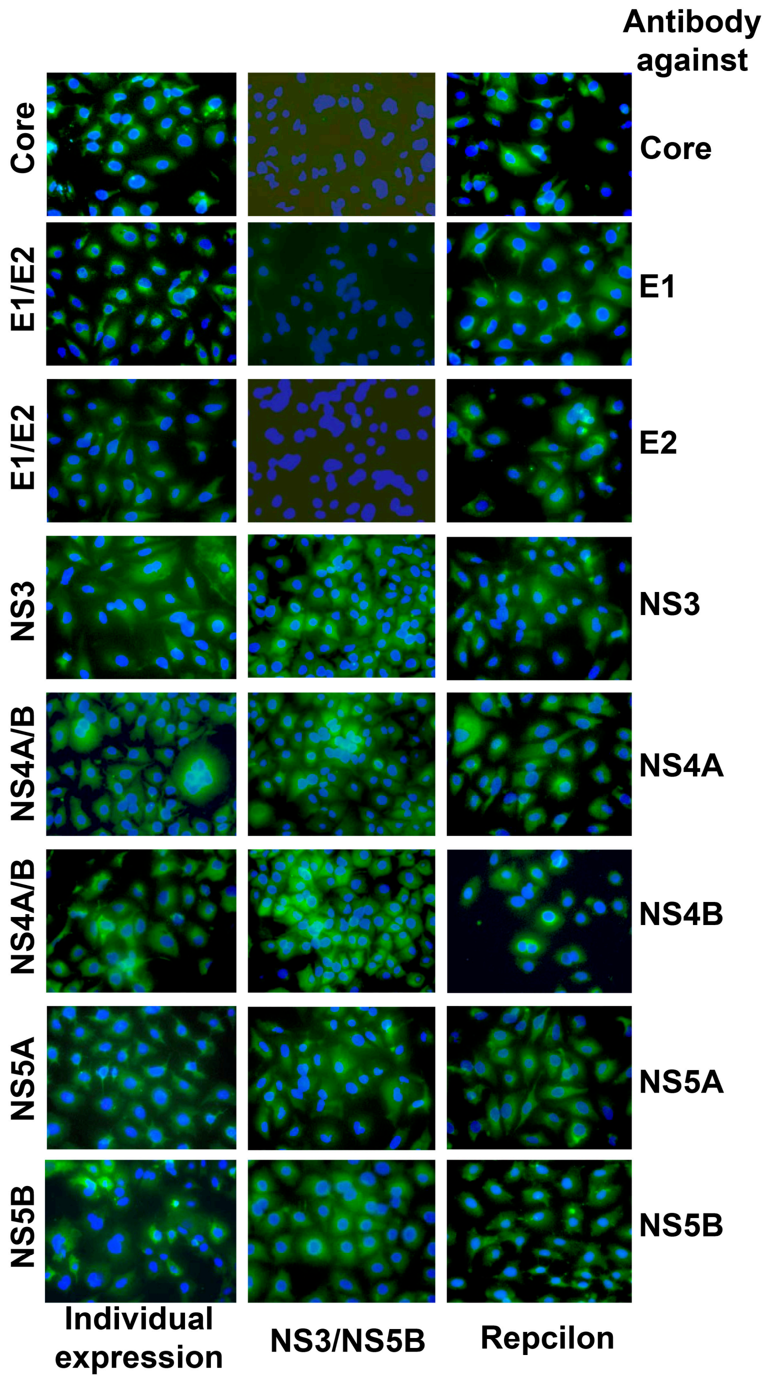

Figure S1. Immunocytochemical staining of HCV protein in cells transfected with plasmids that encoded individual protein (left), NS3-NS5B polyprotein (middle) or harboring HCV replicon (right). The antibody used is listed on the right.

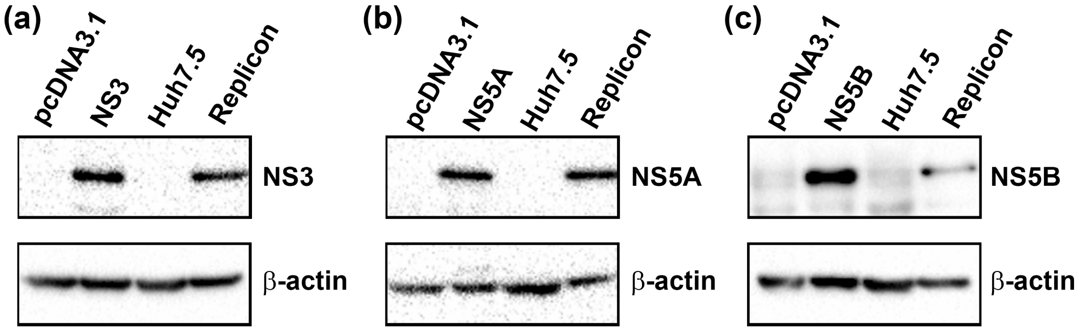

Figure S2. Levels of expression of NS3 (**a**) NS5A (b) and NS5B (c) proteins in cells transfected with plasmids encoding respective individual protein or harboring HCV replicon. The nontransfected Huh7.5 cells and transfected with the empty pcDNA3.1 vector were used as negative controls. The transfected cells were subjected to Western blot analysis 72 h posttransfection.
